# Molecular epidemiology and clinical characteristics of hepatitis delta virus (HDV) infected patients with elevated transaminases in Shanghai, China

**DOI:** 10.1186/s12879-020-05275-1

**Published:** 2020-08-03

**Authors:** Shanshan Wu, Yi Zhang, Yuyan Tang, Ting Yao, Mengjiao Lv, Zhenghao Tang, Guoqing Zang, Yongsheng Yu, Xiaohua Chen

**Affiliations:** Department of Infectious Diseases, Shanghai Jiao Tong University Affiliated Sixth People’s Hospital, Shanghai, 200233 China

**Keywords:** Hepatitis Delta virus, Genotype distribution, Next-generation sequencing, RT-nested PCR

## Abstract

**Background:**

Patients coinfected with HBV and hepatitis D virus (HDV) have a greater risk of HCC and cirrhosis. The current study was undertaken to assess HDV genotype distribution and determine clinical characteristics of hepatitis delta virus (HDV) among HBsAg positive individuals in Shanghai.

**Method:**

This retrospective study involved 225 serum samples from HBsAg positive hospitalized patients from October 2010 to April 2013. HDV-specific RT-nested PCR was used to amplify HDV RNA. HDV genotypes were characterized by Next-generation sequencing (NGS), followed by phylogenetic analyses. HDV/HBV co-infected patients and HBV mono-infected patients were compared clinically and virologically.

**Results:**

Out of the 225 HBsAg-positive serum samples with elevated transaminases, HDV-RNA was identified in 11 (4.9%) patients. The HBV loads in the HDV positive group were significantly lower than the HDV negative HBV-infected patients. The aminotransferase enzymes were significantly higher in HDV/HBV co-infected compared to HDV negative patients (*P* < 0.05). Phylogenetic analyses indicated that HDV-2 genotype being the predominant genotype, other HDV genotypes were not observed. HDV/HBV patients were significantly associated with a rather unfavourable clinical outcome.

**Conclusion:**

In summary, the prevalence of HDV infection in patients with elevated transaminases is not low and the predominance of HDV genotype 2 infection in Shanghai. This finding helps us to better understand the correlation of HDV/HBV co-infection. Moreover, Next-generation sequencing (NGS) technologies provide a rapid, precise method for generating HDV genomes to define infecting genotypes.

## Background

More than 240 million people throughout the world are chronically infected with hepatitis B virus (HBV), and approximately 15–25 million are co-infected with hepatitis D virus (HDV), a satellite virus which requires HBV envelope proteins for particle assembly and spread [1]. The HDV virions possess an outer layer containing hepatitis B surface antigens (HBsAg) and host lipid surrounding the inner nucleocapsid that consists of viral RNA. Thus, HDV depends on HBV for its propagation and replication. Moreover, HBV/HDV co-infection leads to the most severe form of viral hepatitis with an accelerated progression to liver fibrosis, cirrhosis and hepatocellular carcinoma.

In some regions of the world with vaccination campaigns against HBV, the prevalence of HDV infection has significantly declined. However, prevalence of HDV infection is higher in areas where HBV infection is endemic, as well as the central Europe because of the immigrant population regions. HDV Genotype 1 is present globally, whereas other HDV genotypes occur in specific geographical regions. Genotype 2 (previously 2a) prevails in Japan, Taiwan and Russia [2, 3]. Genotype 3 which is the most diverged one is exclusively found in Amazon Basin [4]. HDV-4 (previously 2b) is found in Japan and Taiwan, whereas genotypes 5–8 are described in individuals of African origin [5]. China is one of the countries with the highest number of hepatitis B virus infections in the world, however, no nationwide cohort study has been conducted to assess the prevalence of HDV infection.

Usually, the HDV prevalence is commonly described as percentage of anti-HDAg positive individuals in HBsAg-positive patients using commercial ELISA kits. Anti-HDV seropositive specimens still need to be tested for serum HDV RNA. Several commercial anti-HDV tests were used as methods of simultaneous competitive assays. Recently, quantitative microarray antibody capture (Q-MAC) assay was shown closely related with the presence of HDV-RNA (sensitivity, 100%; specificity, 94.3% for Q-MAC assay) [6]. Anti-HDV IgG is the most commonly used tool to screen patients with CHB for concomitant HDV infection or previous infection. Thus HDV RNA is the “gold standard” for the current diagnosis of HDV infection. Quantitative determination of HDV RNA can be used to monitor the response of antiviral therapy and to determine HDV genotype by sequencing HDV RNA positive samples. There is no standard commercial PCR method, which brings great challenges to diagnosis of HDV infection.

Cloning of PCR products and subsequent Sanger dideoxy sequencing technique have been widely used for the genetic analysis, especially in estimating viral populations. However, the two sequencing methods are time-consuming and laborious. Furthermore, preferential selection of deficient viral genomes can bias the results of molecular cloning. Next-generation sequencing (NGS) method had made it possible to generate hundreds of thousands of clonal sequence reads, which provided the potential to reduce the time and complexity for DNA sequencing without the need for cloning. Furthermore, NGS can character genetic diversity with enhanced sensitivity, efficiency, and accuracy.

HBV infection remains a major public health problem in China, with 10–15% of the population being HBsAg positive_._ Molecular epidemiological studies of HDV prevalence and HDV genotypes in HBV endemic areas in China are needed to assess and improve control measures for HBV/HDV co-infection. Therefore, in the study, we investigated the molecular epidemiology and clinical characteristics of HDV infected patients in Shanghai.

## Methods

### Patients

A total of 225 serum samples with positive HBsAg and HBV DNA were collected from patients in Shanghai Jiao Tong University Affiliated Sixth People’s Hospital from October 2010 to April 2013. All patients were seronegative for HCV and HIV antibodies. Patients with other liver disease (drug induced, alcoholic, autoimmune, metabolic) were excluded by serological tests and detailed anamnesis. They had not received any antiviral or immunomodulatory treatment in the preceding 6 months. Anti-HDV IgG was detected by enzyme-linked immunosorbent assay kit (Beijing Beier Biological Engineering Co., Ltd., China). Serum or plasma samples were collected from those patients’ blood and used for biochemical and other laboratory routine procedures. Samples were stored at − 80 °C. Demographic and laboratory data were obtained from the Electronic Medical Record (EMR). All patients were not treated with drugs and did not progress to liver cancer. Ethical approval was obtained from the Human Ethics Committee of Shanghai Jiao Tong University Affiliated Sixth People’s Hospital (No.2016–73).

### Extraction of HDV RNA and first complementary DNA (cDNA) synthesis

Nucleic acid was extracted from 100 μL serum sample using TaKaRa MiniBEST Viral RNA/DNA Extraction Kit (TaKaRa) according to the manufacturer’s instructions and was kept at − 80 °C until further use. About 50 ng of the extracted RNA was reverse transcribed into cDNA using Superscript III reverse transcriptase (Invitrogen) following the manufacturer’s instructions.

### Molecular detection and genotyping of HDV

The HDV-specific nested polymerase chain reaction (PCR) was carried out with first cDNA samples as template to amplify the HDV delta-gene fragment, which included the HDAg open reading frame (ORF) editing site. Briefly, cDNA was amplified in the first PCR reaction using the HDV-specific primers of PCR forward primer and the reverse primer. The specific primers were synthesized as described in reference [7]. The first primer pairs used were HDV850 (5′- CGG ATG CCC AGG TCG GAC C-3′) and HDV1380 (5′- GGA GCW CCC CCG GCG AAG A-3′). The second primer pairs used were HDV-887 (5′-GAG ATG CCA TGC CGA CCC GAA GAG-3′) and HDV-1290 (5′-GAA GGA AGG CCC TCG AGA ACA AGA-3′). First round PCR amplification was employed in a PCR reaction tube volume of 50 μl containing 10 mM concentration of each of the four deoxynucleotides, 10 uM of each outer sense nucleotides and outer anti-sense primers; and 0.5 ul High-Fidelity DNA Polymerases and 2 ul cDNA. In the thermal cycler, firstly the samples were incubated on 98 °C for 2 min then it was followed by 35 cycles consisting of 98 °C for 10 s, 65 °C for 30 s and 72 °C for 15 s [2]. We used the negative and positive controls along with samples in each run. Finally nested PCR products (10 μl) were electrophoresis on a 1% agarose gel. All nested PCR products were sequenced by NGS on the illumina miseq system (Illumina Inc.,San Diego, CA, USA) following the manufacturer’s protocol. BLAST database (http://blast.ncbi.nlm.nih.gov) to compare input sequence with related reference sequences in the HDV database from the Gene Bank of the National Center for Biotechnology Information (NCBI). Genotypes were assigned by comparing with reference sequences. The HDV sequences were then referenced to the BLAST database (http://blast.ncbi.nlm.nih.gov) to determine the genotype.

### Sequence alignments and phylogenetic analyses

Positive samples were sequenced using an automatic sequencer. Sequences were compiled using the BioEdit program, MEGA7 (molecular evolutionary genetics analysis, version 7.0). The following eight prototype HDV-sequences retrieved from the NCBI GenBank were used for alignment and HDV genotyping: (HDV-1: AF098261, AJ000558, AY633627, HM046802,NC001653, KF660600, KF660601, KF660602; HDV-2: KF660599, AF104264, AY261459, MK234594, MK234593; HDV-3: AB037947, AB037948, AB037949; HDV-4: AF018077, AF209859; HDV-5: AM183326, AM183331, JA417551; HDV-6: AJ584847, AM183332; HDV-7: AM183333, JA417541; HDV-8: AM183327, AM183330). The phylogenetic tree reconstruction and the mean value of genetic diversity of DNA sequences were carried out using MEGA 7 software.

### Statistical analysis

All the data was analyzed and the summary statistic was carried out by SPSS version 19.0 (a statistical package). T-test was used to determine the risk factors for acquiring HDV infection. Levels of HBV DNA were expressed as log10 copies/mL. A *P* value < 0.05 were considered significant.

## Results

### Sample characteristics and HDV prevalence

Overall, we analyzed clinical characteristics of 225 patients infected with HBV over six months from October 2010 to April 2013, 159 (70.7%) were females and 66 (29.3%) were males. Results indicated that median age of mono-HBsAg positive patients and HDV-RNA/HBsAg-positive patients were 41.4 ± 13 years and 45.5 ± 14.9 years, respectively (*P* < 0.05). None of the HDV-RNA positive samples was anti-HDV antibodies positive, even all 11 patients were antibody negative. However, 11 of the 225 HBsAg-positive serum samples demonstrated detectable HDV-RNA given a HDV prevalence of 4.9% in our study, which may contribute to underestimating the burden of HDV infection.

### Detection of HDV RNA in HBsAg positive patients by agarose gel electrophoresis

In 225 patients with HBsAg positive, HDV RNA target gene amplification was detected and agar sugar electrophoresis detected 11 cases of HDV RNA positive specimens. Partial nested PCR products (403 bp) were displayed on agarose gel.

### Distribution of HDV genotypes

**Fig. 1:**
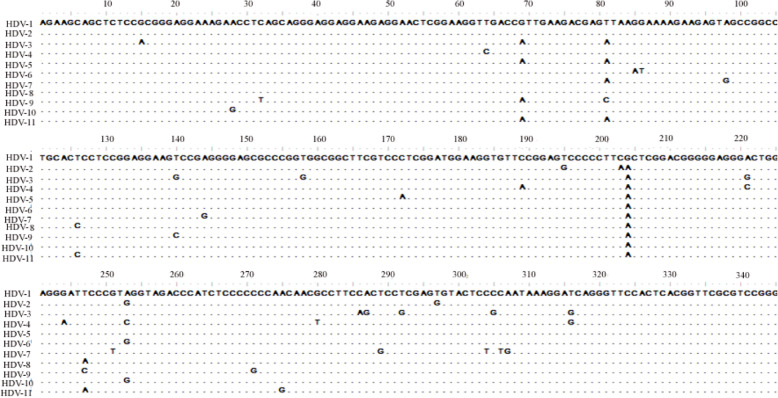
Nucleotide alignment of genomic sequences of HDV isolates

**Fig. 2:**
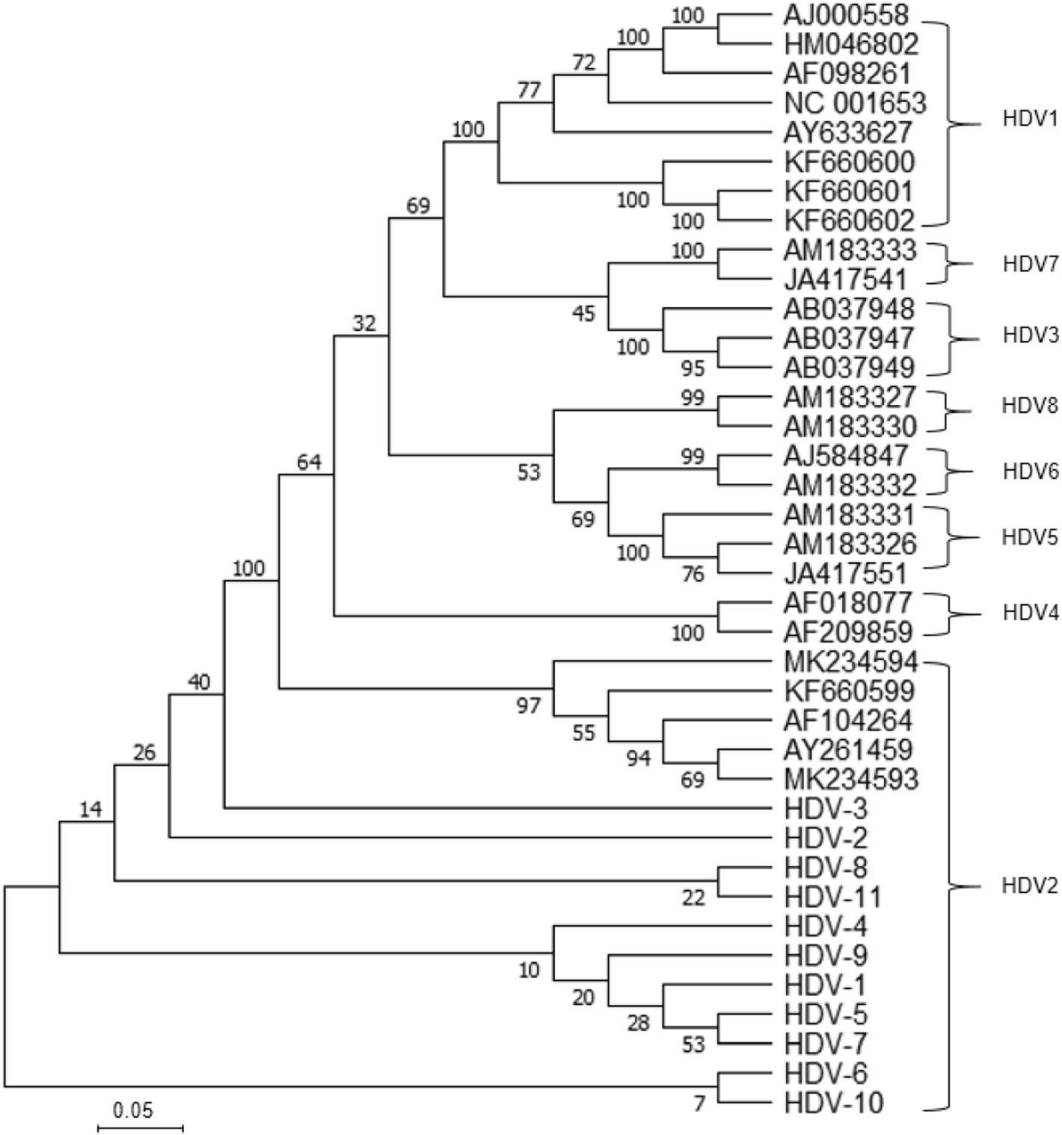
Phylogenetic analysis based on HDV delta-gene sequences. The horizontal branch was drawn in accord with the relative genetic distance. A number of commonly used reference delta-gene sequences for classifying HDV genotypes were also included and are indicated by accession numbers

To determine HDV genotype in Shanghai, we obtained sequence data of HDV RNA-positive samples via amplifying the HDV delta-gene fragment (L-HDAg region) using automatic sequencer. All of the sequences obtained were compiled using BioEdit, CLUSTX. Distribution of HDV genotypes based on phylogenetic analysis is shown in Fig. 1 and Fig. 2. Phylogenetic analysis was performed using sequences of the HDV amplicons and analysis showed that all analyzed HDV genotypes belong to HDV-2 strain. Other HBV genotypes were not detected in this study population. The HDV strains were also clustered in the Asian clade.

### HDV and chronic HBV infection clinical outcomes

The clinical and subclinical characteristics of the patients with or without HDV infection were presented in Table 1. The HBV DNA loads in the HBV/HDV coinfected group were significantly lower in comparison to the HDV negative group (3.85 log10 copies/mL vs*.* 5.16 log10 copies/mL, *P* < 0.05). And the aspartate aminotransferase was significantly higher in HDV positive compared to HDV negative HBV-infected patients (*P* < 0.05).

**Table 1:** Clinical characteristics of the HBsAg positive patients

characteristics	HDV/HBV (*n* = 11)	HBV (*n* = 214)	P value
Age (yr)	45.45 ± 14.94	41.39 ± 13.01	.026
Male:Female	6/5	60/154	.885
ALT(IU/l)	1485.65 ± 935.53	768.58 ± 824.03	.006
AST (IU/l)	977.56 ± 939.71	424.29 ± 474.41	.000
HBV-DNA (log10 copies/mL)	3.852.11	5.16 ± 2.00	.038
TBIL (μmol/L)	80.71 ± 68.13	64.36 ± 87.19	.942
DBIL (μmol/L)	73.07 ± 71.03	43.52 ± 63.15	.446
AKP(U/L)	151.30 ± 66.58	114,01 ± 63.52	.442
γ-GT(U/L)	196.34 ± 119.76	144.93 ± 122.32	.064

*IU* international unit; data are given as median with range, *ALT* alanine aminotransferase, *AST* aspartate aminotransferase, *TBIL* total bilirubin, *DBIL* direct bilirubin, *AKP* alkaline phosphatase, *γ-GT* γ-glutamyl-transferase. *P* values are presented for comparisons between HBV-HDV positivity vs. HBV monopositivity

## Discussion

HDV, the defective satellite RNA virus, was first discovered 41 years ago by Mario Rizzetto that can only assemble and propagate in patients with hepatitis B virus (HBV). Most countries of the Asian-Pacific region are known to be endemic for HBV. Understanding the prevalence of HDV and its genotypes, which are now identified into eight major genotypes, is very important as part of a molecular clue for distribution of HDV.

The distribution of HDV is still present worldwide, and with a higher incidence in Amazonas, Mongolia, Kiribati, and in Asian countries [8]. In China, a large reservoir of HBV infection, testing for HDV is limited and the burden of HDV is likely underestimated. In a study from Taiwan, high-risk populations like human immunodeficiency virus (HIV) infection and injection drug users (IDUs) had higher prevalence of HDV infection, contrasting with the HBsAg positive subjects [2]. Our research is the first one to describe the molecular epidemiology of HDV in Shanghai, a city of mainland China, which is a very important study as it describes the HBV/HDV co-infection using molecular methods. Our study showed that the epidemiology of HDV infection among the HBsAg positive subjects from Shanghai area remained low in this study (4.9%). HDV-1, HDV-2 and HDV-4 are found in China [9]. Surprisingly, our current study showed HDV-2 is the predominant distribution of HDV genotype. However, larger sample size and wider area studies are needed to confirm this distribution of HDV genotype in China.

Several studies had shown that HBV/HDV co-infection could suppress HBV replication with lower levels of HBV viraemia seen in patients positive with HBsAg. In line with mentioned studies, our results indicated that the levels of HBV-DNA were suppressed in patients with HBV/HDV co-infection, suggesting inhibitory effects of HDV on HBV. The potential virological mechanism of inhibition HBV by HDV may be HDV proteins p24 and p27 inhibitor HBV enhancer [10]. Importantly, higher ALT/AST levels in HBV/HDV co-infection patients were detected in our study, which indicated an increased liver damage.

A limitation of our study is that we don’t have sufficient patients. We collected only 225 serum samples for HDV RNA detection and all samples were tested for anti-HDV IgG antibodies. It can be hypothesized that the current commercial ELISA kits have insufficient sensitivity and result in a low detection rate of antibodies. Repeated testing could improve the detection rate of the virus, however RNA is easy to degrade and therefore reduces HDV detection rate. So it’s very difficult to get accurate data of HDV infection rate without a reliable detection method. Meanwhile, our present study of the prevalence of HDV infection mainly focusing on Shanghai area might not be representative for whole China. However, slight variations can significantly modify the national data. Our study provides new insights into the prevalence and genotype distribution in Shanghai. Moreover, further studies are needed to understand the molecular epidemiology of HDV in different areas in China.

## Conclusions

In conclusion, our study showed that the prevalence of HDV infection in patients with elevated transaminases is not low and the predominance of HDV genotype 2 infection in Shanghai. This finding also helps us to better understand the correlation of HBV/HDV co-infection.

## Data Availability

The datasets generated and/or analyzed during the current study may be made available from the corresponding authors on reasonable request.

## Abbreviations

HDV: Hepatitis D virus
HBV: Hepatitis B virus
ALT: Alanine aminotransfease
AST: Aspartate transaminase
PCR: Polymerase chain reaction
HIV: Human immunodeficiency virus
HCC: Hepatocellular carcinoma
NGS: Next generation sequencing
IUD: Injection drug users
HBsAg: Hepatitis B surface antigen
DBIL: Direct bilirubin
AKP: Alkaline phosphatase
γ-GT: γ-glutamyl-transferase
